# Integrated Analysis of miRNAs and Gene Expression Profiles Reveals Potential Biomarkers for Osteoarthritis

**DOI:** 10.3389/fgene.2022.814645

**Published:** 2022-06-17

**Authors:** Zhen Li, Zhenyue Chen, Xiaotan Wang, Zehui Li, He Sun, Jinqiang Wei, Xianzhong Zeng, Xuewei Cao, Chao Wan

**Affiliations:** ^1^ The Second Clinical Medical College of Guangzhou University of Chinese Medicine, Guangzhou, China; ^2^ The First Clinical Medical College of Guangzhou University of Chinese Medicine, Guangzhou, China; ^3^ The First Clinical School, Shandong University of Traditional Chinese Medicine, Jinan, China; ^4^ Department of Orthopaedic Surgery, Guangdong Provincial Hospital of Traditional Chinese Medicine, Guangzhou, China; ^5^ School of Biomedical Sciences, Faculty of Medicine, The Chinese University of Hong Kong, Hong Kong, Hong Kong SAR, China

**Keywords:** osteoarthritis, differentially expressed genes, miRNA sequencing, weight co-expression network, ceRNA network, immune infiltration

## Abstract

**Purpose:** Currently, the early diagnosis and treatment of osteoarthritis (OA) remain a challenge. In the present study, we attempted to explore potential biomarkers for the diagnosis and treatment of OA.

**Methods:** The differentially expressed genes (DEGs) were identified based on three mRNA datasets of synovial tissues for OA patients and normal controls downloaded from the Gene Expression Omnibus (GEO) database. Furthermore, Gene Ontology (GO) and Kyoto Encyclopedia of Genes and Genomes (KEGG) pathway enrichment analysis were used for evaluating gene function related categories. Then, miRNA sequencing was performed for differentially expressed miRNAs’ identification. Finally, weighted gene co-expression network analysis (WGCNA) was performed for genes detected by the three mRNA datasets and a competing endogenous RNA (ceRNA) network with DEGs and differentially expressed microRNAs (miRNAs) was constructed for central genes identification. In addition, the relationship between central gene expression and immune infiltration was analyzed, and the candidate agents for OA were predicted based on the Connectivity Map database. Quantitative RT-PCR (qRT-PCR), Western blotting analysis, and immunofluorescent staining were performed to validate the expression levels of differentially expressed miRNAs and differentially expressed target genes in normal and OA tissues and chondrocytes. MiRNA–mRNA network was also validated in chondrocytes *in vitro*.

**Results:** A total of 259 DEGs and 26 differentially expressed miRNAs were identified, among which 94 miRNA–mRNA interactions were predicted. The brown module in WGCNA was most closely correlated with the clinical traits of OA. After overlapping the brown module genes with miRNA–mRNA pairs, 27 miRNA–mRNA pairs were obtained. A ceRNA network was constructed with 5505 lncRNA–miRNA–mRNA interactions. B-cell translocation gene 2(BTG2), Abelson-related gene (ABL2), and vascular endothelial growth factor A (VEGFA) were identified to be the central genes with good predictive performance, which were significantly correlated with immune cell infiltration in OA, reflected by declined activated dendritic cells (aDCs), and elevated contents of B cells, macrophages, neutrophils, and T helper cells. Anisomycin, MG-132, thapsigargin, and lycorine were predicted to be the potential candidate agents for OA intervention. *In vitro*, the expression levels of differentially expressed miRNAs and biomarkers identified in the present study were consistent with the results obtained in normal or OA knee cartilage tissues and chondrocytes. Furthermore, BTG2 was identified to be negatively regulated by miR-125a-5p.

**Conclusion:** BTG2, ABL2, and VEGFA can be regarded as potential predictive and treatment biomarkers for OA, which might guide the clinical therapy of OA.

## Introduction

Osteoarthritis (OA) is the most common type of arthritis characterized by the loss of articular cartilage under the joints and bones. Its main symptoms include joint pain, swelling, and stiffness, which severely affect physical function and the quality of life. OA typically affects the joints, but it also shows a relationship with cardiovascular diseases, such as myocardial infarction (Schieir et al., 2017) and coronary heart disease (Chung et al., 2016). It is estimated that OA affects 242 million people worldwide and the frequency of OA in women is higher than in men (Breedveld, 2004). OA is the primary cause of joint damage and irreversible disability. Thus, the early diagnosis and aggressive treatment management of OA are necessary for improving the prognosis of this disease.

Emerging evidence has revealed that biomarker identification facilitates the improvement of diagnosis and treatment of OA (Watt, 2018). The level of serum superoxide dismutase 2 (SOD2) is elevated in OA patients indicating OA related oxidative stress and has been suggested as the candidate diagnostic biomarker (Fernandez-Moreno et al., 2011). The baseline collagen type II cleavage neoepitope (C2C) level in serum predicts the risk of cartilage damage in OA (Poole et al., 2016). C-reactive protein (CRP) is one of the inflammatory markers strongly associated with the incidence and progression of OA (Saberi et al., 2016). The circulating lipopolysaccharide associated with systematic inflammation is the indicator for an abundance of activated synovial macrophage abundance and osteophyte severity (Watt, 2018). However, more biomarkers are indeed needed to more accurately predict the pathological process of OA so as to achieve the goal of multi-targeted therapy for OA.

The development of next-generation technologies such as large-scale sequencing and multianalyte assay has recently facilitated the discovery of a slew of potential biomarkers for OA (Cong et al., 2017; Xu et al., 2019; Zhang et al., 2019; Hong et al., 2020). For example, Li et al. (2017), identified several differentially expressed genes, including a disintegrin and metalloproteinase 8 (Adam8), arginase 1 (Arg1) and microRNAs (miRNAs) such as miRNA-200b, which might play key roles in the pathogenesis of mice OA based on microarray analysis. Furthermore, functional disruption of collagen in cartilage has been shown to be a very important contributing factor to OA. Quantitative detection of collagen synthesis may facilitate early identification and prediction of OA (Zhang et al., 2019). However, none of these biomarkers is specific, which makes diagnosis and treatment of OA still a challenge. In the present study, we added our own miRNA microarray data and combined them with gene expression datasets deposited in the Gene Expression Omnibus (GEO) database to perform a comprehensive bioinformatic analysis of potential biomarkers for OA. Furthermore, a new ceRNA network was constructed, and drug prediction analysis for OA was performed. In addition, select differentially expressed miRNAs and their target genes were further validated by *in vitro* experiments. The promising potential biomarkers with predictive function in the pathogenesis and treatment of OA identified in this study are expected to guide novel interventions of OA.

## Methods

### Data Downloads

**FIGURE 1:**
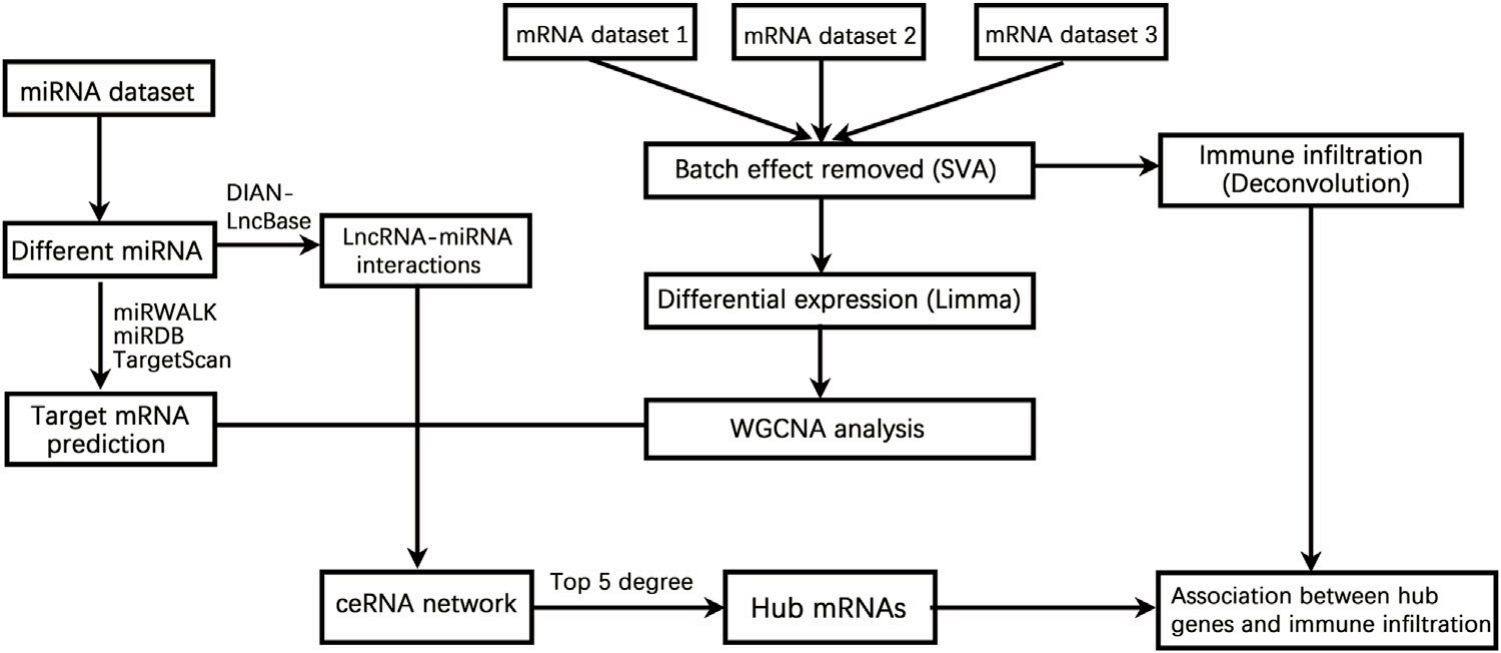
A flow chart showing the steps involved in the bioinformatic analysis of datasets obtained from GEO. The differentially expressed genes (DEGs) were identified based on three mRNA datasets. miRNA sequencing was performed for the identification of differentially expressed miRNAs. Weighted gene co-expression network analysis (WGCNA) was performed for genes detected using three mRNA datasets, and a ceRNA network with DEGs and differentially expressed microRNAs (miRNAs) was constructed for central genes identification. The relationship between central gene expression and immune infiltration was analyzed, and the candidate agents or small molecules for potential OA therapy were predicted based on the Connectivity Map database.

Three mRNA datasets of joint synovial samples [accession numbers GSE 1919 (Ungethuem et al., 2010), GSE55235 (Woetzel et al., 2014), and GSE55457 (Woetzel et al., 2014) ] were available from NCBI GEO database. The dataset of GSE1919 included joint synovial tissues of five OA patients and five normal donors. The downloaded GSE55235 dataset contained joint synovial tissue samples from ten OA patients and ten healthy joints of individuals who suffered from fatal accidents. From the GSE55457 dataset, the mRNA data of synovial membranes from ten OA patients and ten normal donors were retrieved. The txt format series matrix files were downloaded, and the batch effect was removed by the surrogate variable analysis (SVA) algorithm. The differentially expressed genes (DEGs) between OA and normal controls were identified by the limma package (version 3.46.0) (Smyth et al., 2005; Ritchie et al., 2015). *p* < 0.05 and |logFC(fold change)|>1 were set as the cutoff value. The key steps involved in the bioinformatic analysis of datasets obtained from GEO are illustrated in Figure 1.

### Function Enrichment Analysis

Gene Ontology (GO) and Kyoto Encyclopedia of Genes and Genomes (KEGG) pathway enrichment analysis were used for evaluating gene function related categories. DEGs were annotated based on GO and KEGG databases using the ClusterProfiler package in R (version 3.18.1) (Yu et al., 2012). The significantly enriched GO and pathway terms were screened with *p* < 0.05 and *q* < 0.05.

### MiRNA Sequencing and Differential Expression Analysis

Joint synovial tissues from OA patients (five pairs) and normal controls (five pairs) were obtained under surgery in Guangdong Provincial Hospital of Traditional Chinese Medicine (NO. BF 2021-004). Approval was obtained from the ethics committee of Guangdong Provincial Hospital of Traditional Chinese Medicine, and the study procedures complied with the declaration of Helsinki (World Medical Association, 2013). Total RNA was prepared from synovial tissues and purified using the mirVana miRNA isolation kit (Ambion, Austin, TX, United States) according to the manufacturer’s protocols. The quantified RNA was ligated with 3′ and 5′ adaptors. The miRNA-adaptor ligation products were subjected to qRT-PCR amplification. Then, the cDNA library was sequenced on the DNBSEQ platform (Beijing Genomic Institute, Shenzhen, China). The clean reads were aligned to the human reference genome (the Human Genome U95 Set and Human Genome U133A), and the differentially expressed miRNAs were investigated based on the FPKM method by using the limma package. A *p* < 0.05 was considered as statistically significant.

### Weighted Gene Co-Expression Network Analysis

WGCNA facilitates performing weighted correlation network analysis and describing the correlation structure between gene expression profiles, image data, genetic marker data, and proteomics data (Langfelder and Horvath, 2008). The gene co-expression network for DEGs in GSE 1919, GSE55235, and GSE55457 datasets were generated by the WGCNA-R package. The soft threshold was determined using the “sft$powerEstimate” function, and the topological overlap matrix (TOM) was obtained from the adjacency matrix. Hierarchical clustering was performed for TOM to evaluate the structure of the cluster tree. Different branches (colors) of the cluster tree represented different modules.

### Competitive Endogenous RNA Network Construction

The differentially expressed miRNA interacted genes were predicted by miRDB (http://mirdb.org/) (Chen and Wang, 2020), miRTarBase (https://mirtarbase.cuhk.edu.cn/∼miRTar Base/miRTarBase_2022/php/index.php) (Huang et al., 2022), and TargetScan (https://www.targetscan.org/vert_72/) (Agarwal et al., 2015) databases. The DEGs commonly predicted by the three databases were selected for further analysis. The long non-coding RNA (lncRNA)-miRNA interactions were predicted by DIANA-LncBase database (www.microrna.gr/LncBase) (Karagkouni et al., 2020). Based on the correlations between lncRNAs, miRNAs, and mRNAs, a ceRNA network was constructed using Cytoscape software (version3.8.2) (Kohl et al., 2011; Ma et al., 2018). Critical genes were analyzed by connectivity degrees, and the diagnostic performance of interesting genes was validated by receiver operating characteristic (ROC) curve analysis.

### Correlation Analysis of Critical Genes With Immune Infiltration

Based on gene expression profiles of different OA samples, the proportions of 28 types of immune cells were deduced by the single-sample Gene Set Enrichment Analysis (ssGSEA) algorithm and the relative contents of immune cells were depicted by the “vioplot” package (version 0.3.7) (Hu, 2020). The interplay between different types of immune cells was analyzed by the “corrplot” package (version 0.92) (Zhang et al., 2021). The correlation between gene expression level and immune cell proportion was investigated using Spearman correlation analysis. A *p* < 0.05 was considered as statistically significant.

### Drug Small Molecule Prediction

The Connectivity Map (CMap) is a publicly available database that collects gene expression profiles perturbed by a series of small molecules in various cell lines (Langfelder and Horvath, 2008). DEGs were divided into upregulated and downregulated ones. The expression profiles of upregulated and downregulated DEGs were compared with those in the CMap database. The relative correlations between query signature and reference gene expression profiles of individual drugs in the CMap database are output as enrichment scores ranging from −1 to 1. When the enrichment scores are closed to 1, it means that the corresponding small molecules can contribute to the progression of the disease; otherwise, the small molecules can reverse the gene signals induced by the disease. Thus, the small molecules with enrichment scores closed to −1 and *p* < 0.05 were selected as the potential candidate drugs for OA therapy.

### Gene Set Enrichment Analysis

GSEA was developed as a method for gene expression profile analysis (Subramanian et al., 2005). The gene sets were pre-ranked based on the differential expression between OA and normal control groups. The ordered gene sets were subjected to GSEA. The number of permutations was set as 1000, and the permutation type was set as phenotype.

### Chondrocyte Isolation and Culture

This study was performed in accordance with the animal ethics standards and with the approval of the animal ethics committee at the Guangzhou University of Chinese Medicine. Chondrocytes were isolated from embryonic 2-week-old male Sprague–Dawley (SD) rats (Laboratory Animal Center of Guangzhou University of Chinese Medicine, Guangzhou, China) according to an established protocol (Zhang et al., 2014). In brief, cartilage tissue samples were trimmed into small pieces and digested in pronase in PBS (2 mg/ml) for an hour and then in DMEM medium (Gibco, Life Technologies, Carlsbad, CA, United States) containing collagenase D (3 mg/ml) (Roche) or collagenase type Ⅱ (2 mg/ml) (Gibco) for 3 h at 37°C in an incubation shaker. Dissociated cells were cultured in DMEM/F12 medium containing 10% fetal bovine serum (FBS; Gibco, Life Technologies, Carlsbad, CA, United States), 100 U ml^−1^ of penicillin, and 100 mg ml^−1^ of streptomycin (Gibco, Life Technologies, Carlsbad, CA, United States). The cells were cultured in DMEM/F12 at 37°C, 5% CO_2,_ and saturated humidity. The medium was changed every 2 days, and non-adherent cells were discarded. All chondrocytes used in this study were between passages 2 and 4. To establish an OA chondrocyte model, chondrocytes were treated with DMEM/F12 containing interleukin (IL)-1β (10 ng/ml, Sigma-Aldrich, St Louis, Missouri, United States).

### Quantitative Real-Time Polymerase Chain Reaction

The qRT-PCR was performed to evaluate the expression levels of differentially expressed miRNAs, including miR-424-5p, miR-125a-5p, and miR-125b-5p and differentially expressed target genes, including BTG2, ABL2, and VEGFA in chondrocytes and knee cartilage tissues of normal and OA patients. OA patients (*n* = 15) and normal individuals (*n* = 15) were included in this study. The tissues from subjects were obtained under surgery with informed consent. This study was approved by the Ethics Committee of Guangdong Provincial Hospital of Traditional Chinese Medicine (NO.BF 2021-004).

The total RNA was extracted from chondrocytes or knee cartilage tissues using an RNAiso Plus Kit (Takara Biotechnology, Dalian, Liaoning, China). The PrimeScript reverse transcription reagent kit (Takara Biotechnology, Dalian, Liaoning, China) was applied for reverse transcription. The qRT-PCR was conducted using a SteponePlus (Applied Biosystems, Foster, CA, United States) PCR instrument. SYBR^®^ Green Premix *Pro Taq* HS qPCR kit Ⅱ (Accurate Biotechnology, Guangzhou, Guangdong, China) was used for qPCR analysis. The PCR reaction volumes contained 2 μl of cDNA solution, 10 μl of 2 × SYBR^®^ Green *Pro Taq* HS Premix Ⅱ, 0.8 μl of PCR forward primer and 0.8 μl of reverse primer (both at 10 μM) and RNase free water up to 20 μl. With U6 selected as an internal reference, the mRNA expression was detected using the 2^−ΔΔCt^ method. The primer sequences are shown in Table 1. The universal primers in the kit are used as miRNA downstream primers.

**TABLE 1:** Primer sequence list.

Protein-coding genes	Primer sequences
miR-424-5p	5′-CGC​CAG​CAG​CAA​TTC​ATG​TTT​TGA​A-3′
miR-125a-5p	5′-TCC​CTG​AGA​CCC​TTT​AAC​CTG​TGA-3′
miR-125b-5p	5′-TCT​CCC​TGA​GAC​CCT​AAC​TTG​TGA-3′
U6	F:5′-GCTTCGGCAGCACATATACTAAAAT-3′
U6	R:5′-CGCTTCACGAATTTGCGTGTCAT-3′
BTG2-F	F:5′-GCGTGAGCGAGCAGAGGCTT-3′
BTG2-R	R:5′-GGCTGGCCACCCTGCTGATG-3′
ABL2-F	F:5′-GTGATGAGACTACTGCAGCATCC-3′
ABL2-R	R:5′-CGGTTACCGTGTCGTCCATGAT-3′
VEGFA-F	F:5′-TATTCAGCGG ACTCACCAGC-3′
VEGFA-R	R:5′-AACCAACCTCCTCA AACCGT-3′
GAPDH-F	F:5′-ACCCAGAAGACTGTGGATGG-3′
GAPDH-R	R:5′-GAGGCAGGGATGATGTTCTG-3′

### Western Blotting

The Western blot analysis was performed to detect the protein expression of differentially expressed target genes, including BTG2, ABL2, and VEGFA, in chondrocytes and knee cartilage tissues of normal and OA patients. Total protein from each sample was extracted using Radio Immunoprecipitation Assay (RIPA) lysis buffer (Gibco, Grand Island, NY, United States). Subsequently, 20 μg total proteins from each sample was resolved by 10% SDS-PAGE and transferred onto polyvinylidene difluoride (PVDF, Millipore, Billerica, MA, United States) membranes. After being blocked with 5% skim milk for 90 min at room temperature (RT), the membranes were then incubated with the primary antibody solution overnight at 4°C. Subsequently, the membranes were incubated with a horseradish peroxidase (HRP)–conjugated secondary antibody (1:1000) for 90 min at RT. The protein bands were visualized *via* a ChemiDoc™ MP Imaging System (Bio-Rad). GAPDH was used as an internal reference. Primary antibodies used in this experiment were from the following source: Rabbit anti-BTG2 (1:1000), mouse anti-ABL2 (1:1000), rabbit anti-VEGFA (1:1000), and GAPDH (1:5000). All primary antibodies were purchased from Invitrogen (Invitrogen inc., Carlsbad, CA, United States).

### Immunofluorescent Staining

For immunofluorescence, chondrocytes were fixed with 4% PFA for 30 min and permeabilized in 0.3% Triton X-100 at RT for 30 min. Subsequently, the cells were blocked with 10% normal horse serum at RT for 1 h and then incubated with primary antibodies overnight at 4°C. Next, the primary antibodies were conjugated with Alexa Fluor 488/568 goat anti-rabbit or mouse antibodies (1:300) for identification, and then the nucleus was counter-stained with DAPI. Finally, the slides were mounted and observed under a fluorescence microscope (Olympus IX73). Primary antibodies used in this study were as follows: Rabbit anti-BTG2 (1:1000), mouse anti-ABL2 (1:1000), and rabbit anti-VEGFA (1:1000). All antibodies were purchased from Invitrogen (Invitrogen inc., Carlsbad, CA, United States).

### Luciferase Reporter Assay and Chondrocytes Transfection

The target gene of miR-125a-5p was predicted by the online software Targetscan 7.1 (www.targetscan.org/vert_71/). DNA fragments containing the miR-125a-5p binding site in the 3′-untranslated regions (3′-UTR) of BTG2 and fragments containing the mutant miR-125a-5p binding site were separately inserted into the luciferase reporter gene plasmid to obtain the BTG2-WT (wild type) and BTG2-MUT (mutant type) plasmids, respectively. Next, the miR-125a-5p mimics and BTG2-WT/BTG2-MUT plasmids were co-transfected into 293T cells, and the luciferase activities of the transfected cells were detected using luciferase assay. In addition, 293T cells were co-transfected with negative control (NC) oligonucleotide and BTG2-WT/BTG2-MUT plasmids as control. Chondrocytes were seeded onto a six-well plate at a density of 2 × 10^5^ cells/well 2 days prior to transfection. When growing at a confluence of 70%–80%, chondrocytes were transfected with miR-125a-5p mimics, inhibitor, and their negative control (RIBO Biotech Company, Guangzhou, Guangdong, China) using the Lipofectamine 2000 reagent, respectively. At 48 h post-transfection, Western blotting and qRT-PCR were performed to evaluate the effect of cell transfection.

### Statistical Analysis

The SPSS 20.0 software (SPSS, Chicago, IL, United States) was applied for statistical analyses. Results are presented as means ± standard deviations (SDs). Multiple groups were compared using one-way ANOVA, while the differences between two groups were analyzed using an unpaired Student’s *t*-test. A *p* < 0.05 was considered statistically significant.

## Results

### Identification of Differentially Expressed Genes of Synovial Tissues of Osteoarthritis Patients Compared With Normal Controls

**FIGURE 2:**
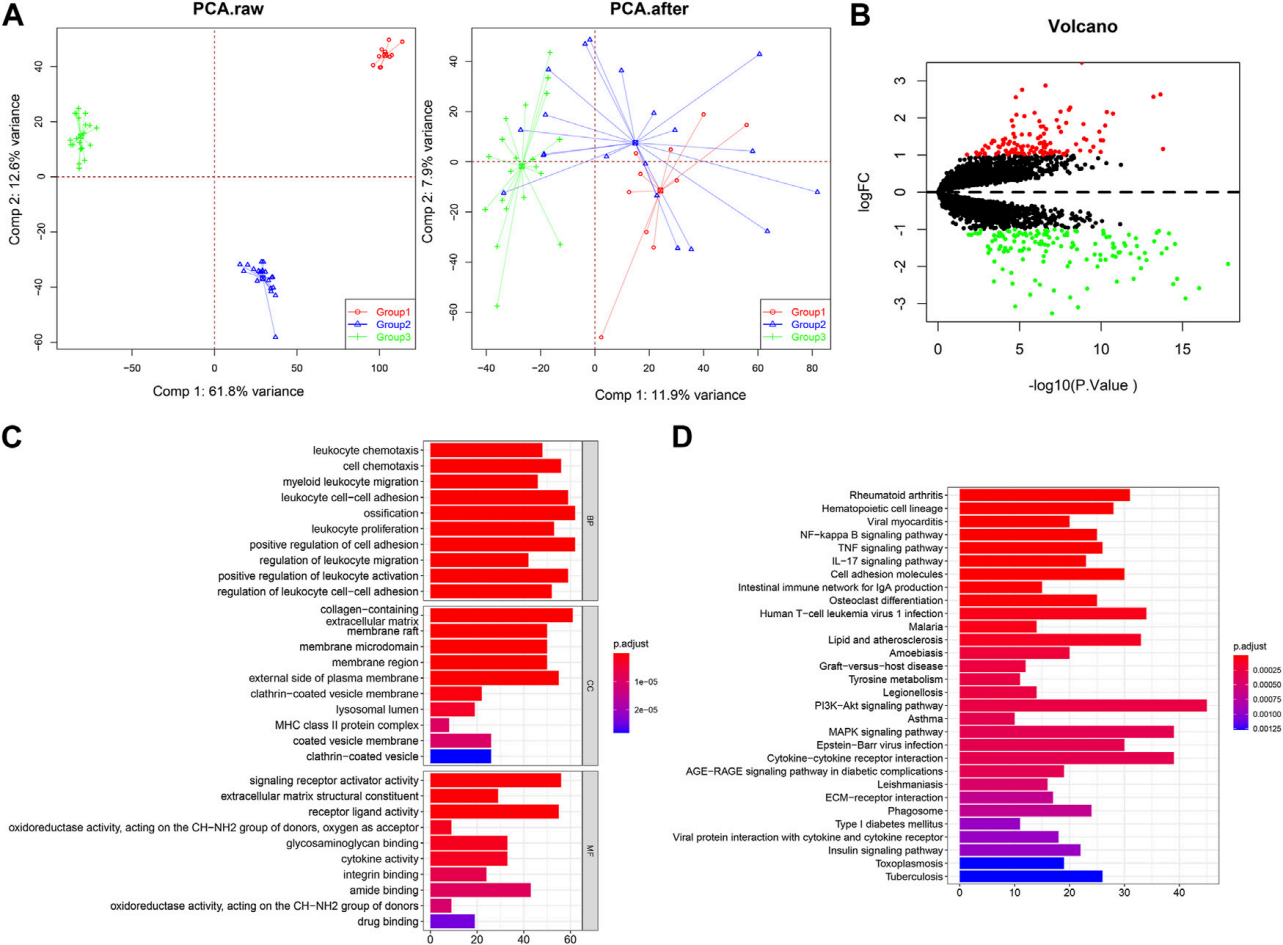
Identification of DEGs in synovial tissues of OA patients compared with normal controls. **(A)** The samples distribution before and after batch effects removal. PCA, principal component analysis. **(B)** Volcano plot for 259 DEGs based on three groups of mRNA datasets. **(C)** GO enrichment analysis of DEGs. **(D)** The significantly enriched pathways by DEGs.

Three gene expression datasets of synovial tissues were downloaded from the GEO database. For data integration, the batch effects were corrected by surrogate variable analysis (SVA). Before batch effects removal, the samples in different batches were separately clearly, while SVA adjustment brought the samples to a single batch (Figure 2A), indicating the batch effects were effectively removed. Then, the DEGs of three groups of OA patients compared with normal controls were identified by the limma package. Results showed that 259 genes were differentially expressed, including 141 upregulated genes and 118 downregulated genes (Figure 2B). Functional analysis indicated that DEGs were closely related to leukocyte chemotaxis, proliferation, migration, and adhesion related biological processes and significantly enriched in tumor necrosis factor alpha (TNF) signaling and Interleukin (IL)-17 signaling pathways (Figures 2C and D).

### Competing Endogenous RNA Network and Identification of Critical Genes

**FIGURE 3:**
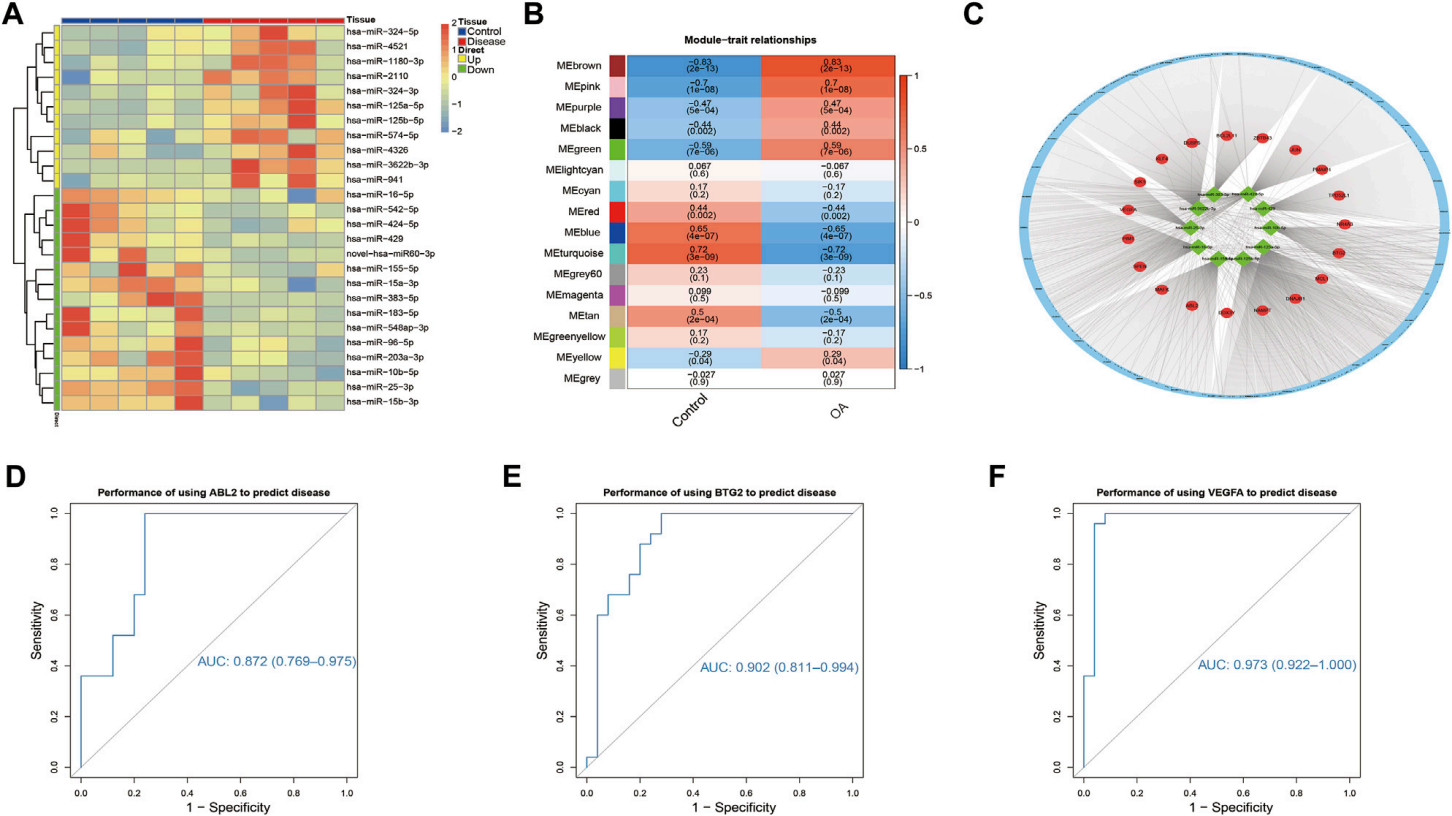
ceRNA network construction and the central genes. **(A)** Heatmap of the differentially expressed miRNAs. The expression profiles of miRNAs were significantly different between OA and control samples. **(B)** The relationships between WGCNA modules and clinical traits. **(C)** ceRNA network constructed using 10 differentially expressed miRNAs and 19 DEGs. Red circles represent DEGs, and green squares represent differentially expressed miRNAs. **(D–F)** The ROC curves for the predictive performance of ABL2, BTG2, and VEGFA for OA.

By miRNA sequencing, we obtained 26 differentially expressed miRNAs (Figure 3A). According to TargetScan, miRDB, and miRTarBase, there were 1219 miRNA–mRNA interactions mapped to differentially expressed miRNAs. After overlapped with DEGs, 94 miRNA–mRNA interaction pairs were collected. The genes detected in three datasets were subjected to WGCNA. The weight co-expression network was classified into 16 modules, including black (*n* = 194), blue (*n* = 957), brown (*n* = 923), cyan (*n* = 182), green (*n* = 222), green-yellow (*n* = 131), grey (*n* = 4), grey60 (*n* = 65), light cyan (*n* = 80), magenta (*n* = 273), pink (*n* = 181), purple (*n* = 155), red (*n* = 198), tan (*n* = 126), turquoise (*n* = 1021), and yellow (*n* = 288). The brown module was most significantly correlated with clinical traits (cor = 0.83, p = 2e−13) (Figure 3B). Thus, the genes in the brown module were overlapped with 94 miRNA–mRNA pairs and finally 27 miRNA–mRNA interactions were obtained. In parallel, 452,052 lncRNA–miRNA interactions were predicted by DIANA-LncBase database. Finally, a ceRNA network was constructed with 5,505 lncRNA–miRNA–mRNA interactions (Figure 3C). B-cell translocation gene 2 (BTG2), Abelson-related gene (ABL2), and vascular endothelial growth factor A (VEGFA) were the key mRNAs with highest node degrees and showed relatively good predictive value for OA [BTG2-AUC: 0.902 (0.811–0.994), ABL2-AUC: 0.872 (0.769–0.975), and VEGFA-AUC: 0.973 (0.922–1.000)] (Figures 3D–F).

### The Correlation Between Critical Gene Expression and Immune Infiltration

**FIGURE 4:**
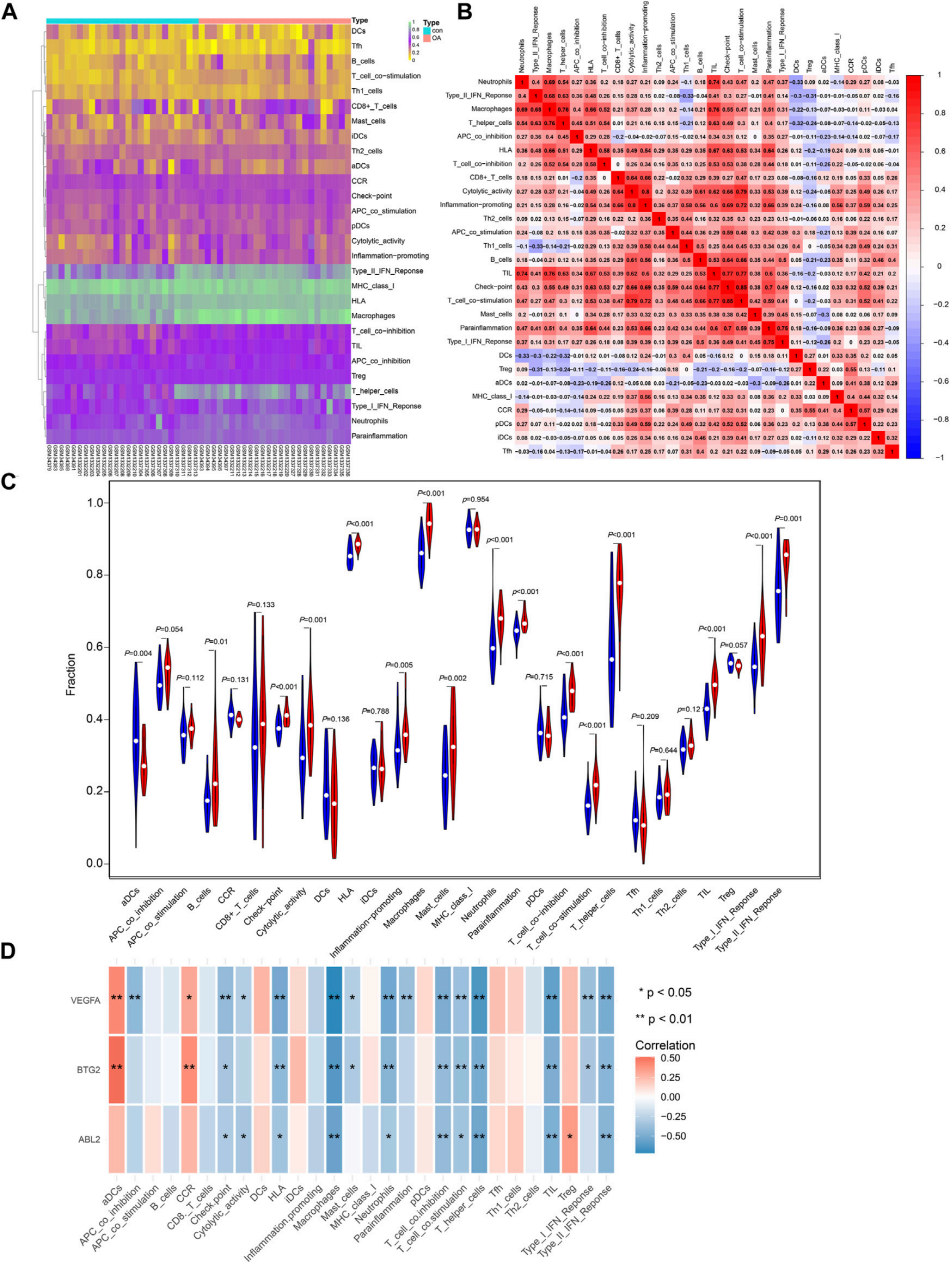
The relationship between critical gene expression and immune infiltration. **(A)** The heatmap of the content for different types of immune cells in OA and control samples. **(B)** The relationship between different immune cells. **(C)** The difference in immune cell content between OA and normal samples. **(D)** The correlation of VEGFA, BTG2, and ABL2 with the content of different immune cells.

The immune cell content in each sample was predicted (Figure 4A), and the correlation between different types of immune cells was depicted in Figure 4B. In addition, the content of activated dendritic cells (aDCs) was remarkably declined in OA patients, while the contents of B cells, check-point, cytolytic activity, HLA, inflammation-promoting, macrophages, mast cells, neutrophils, parainflammation, T cell co-inhibition, T cell co-stimulation, T helper cells, tumor-infiltrating lymphocytes (TIL), Type I IFN response, and Type II IFN response were obviously elevated (Figure 4C). The expression of BTG2, ABL2, and VEGFA was mostly correlated with the different types of immune cells (Figure 4D).

### Candidate Small Molecules Identification

**FIGURE 5:**
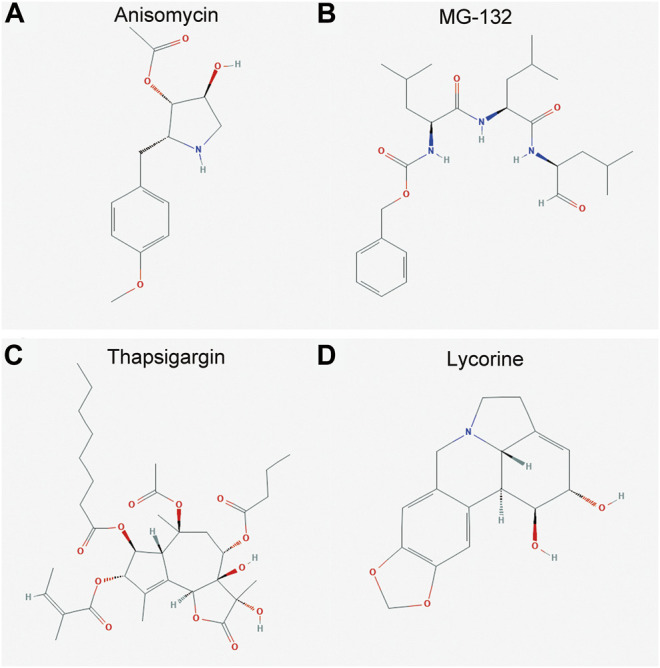
Molecular structure of the candidate agents for OA. **(A–D)** The molecular structure for anisomycin, MG-132, thapsigargin, and lycorine, respectively. The DEGs induced expression profiles in OA were compared with that perturbed by small molecules in the CMap database. Anisomycin **(A)**, MG-132 **(B)**, thapsigargin **(C)**, and lycorine **(D)** induced expression profiles elicited a highly negative correlation with that of the DEGs.

The DEGs induced expression profiles in OA were compared with that perturbed by small molecules in the CMap database. The top 20 small molecules that showed a significant correlation with query signatures are listed in Table 2. Anisomycin, MG-132, thapsigargin, and lycorine induced expression profiles elicited a highly negative correlation with that of DEGs (Figure 5), suggesting that these molecules may be considered as potential candidates for the intervention of OA and as yet deserve further investigations.

**TABLE 2:** Top 20 small molecules predicted by CMap database.

Rank	CMap name	Mean	*n*	Enrichment	*p*
1	doxorubicin	0.862	3	0.999	0
2	H-7	0.871	4	0.999	0
3	alsterpaullone	0.718	3	0.996	0
4	GW-8510	0.725	4	0.994	0
5	anisomycin	−0.842	4	−0.994	0
6	thapsigargin	−0.713	3	−0.987	0
7	digitoxigenin	−0.592	4	−0.976	0
8	lasalocid	−0.529	4	−0.935	0
9	helveticoside	−0.593	6	−0.884	0
10	15-delta prostaglandin J2	−0.33	15	−0.603	0
11	tanespimycin	0.246	62	0.381	0
12	mitoxantrone	0.665	3	0.986	0.00004
13	MG-262	−0.662	3	−0.977	0.00004
14	podophyllotoxin	−0.472	4	−0.911	0.0001
15	lanatoside C	−0.572	6	−0.789	0.00018
16	gossypol	−0.482	6	−0.78	0.0002
17	lycorine	−0.679	5	−0.849	0.00024
18	mycophenolic acid	0.55	3	0.94	0.00028
19	vincamine	−0.31	6	−0.765	0.00032
20	STOCK1N-35696	−0.645	2	−0.983	0.00062

### Significant Pathways Involved With Critical Genes

**FIGURE 6:**
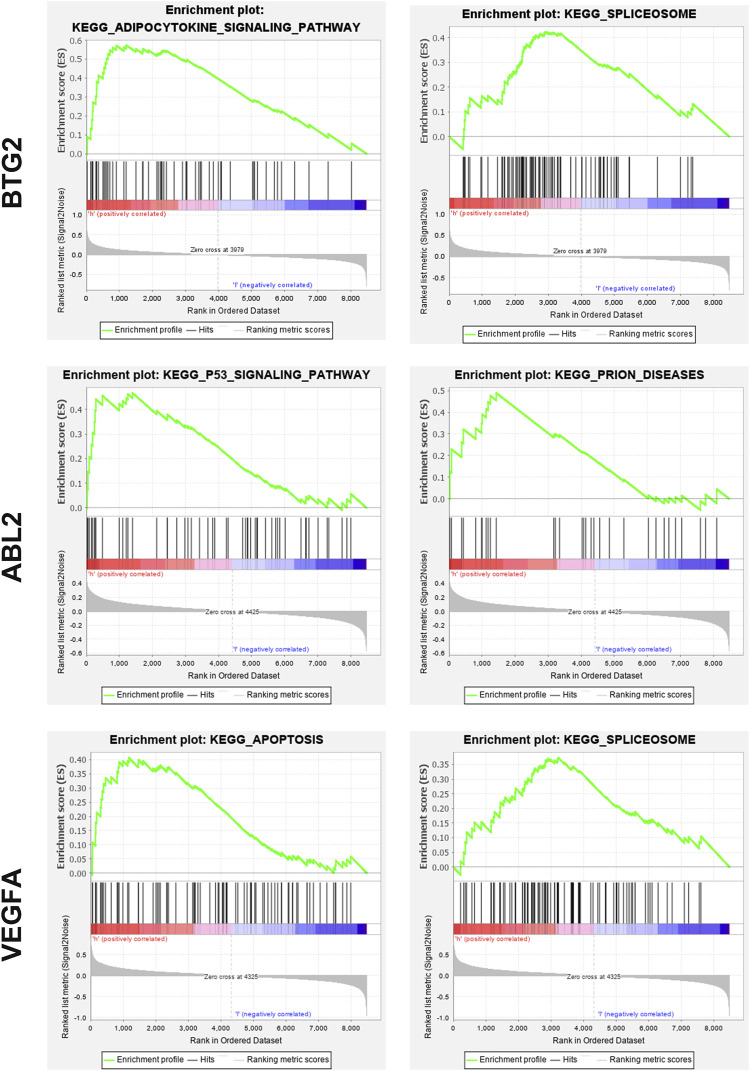
Significant pathways implicated with BTG2, ABL2, and VEGFA. Based on GSEA, BTG2 was mainly involved in KEGG spliceosome and adipocytokine signaling pathways. ABL2 was significantly enriched in KEGG P53 signaling and prion diseases related pathways. VEGFA was closely related to KEGG apoptosis and spliceosome pathways.

We next examined the major signaling pathways in association with the critical genes identified earlier. Based on GSEA, BTG2 was mainly involved in KEGG spliceosome and adipocytokine signaling pathways. ABL2 was significantly enriched in KEGG P53 signaling and prion diseases related pathways. VEGFA was closely related to KEGG apoptosis and spliceosome pathways (Figure 6).

### Expression Levels of Differentially Expressed miRNAs and Target Genes in Normal and Osteoarthritis Tissues and Chondrocytes

**FIGURE 7:**
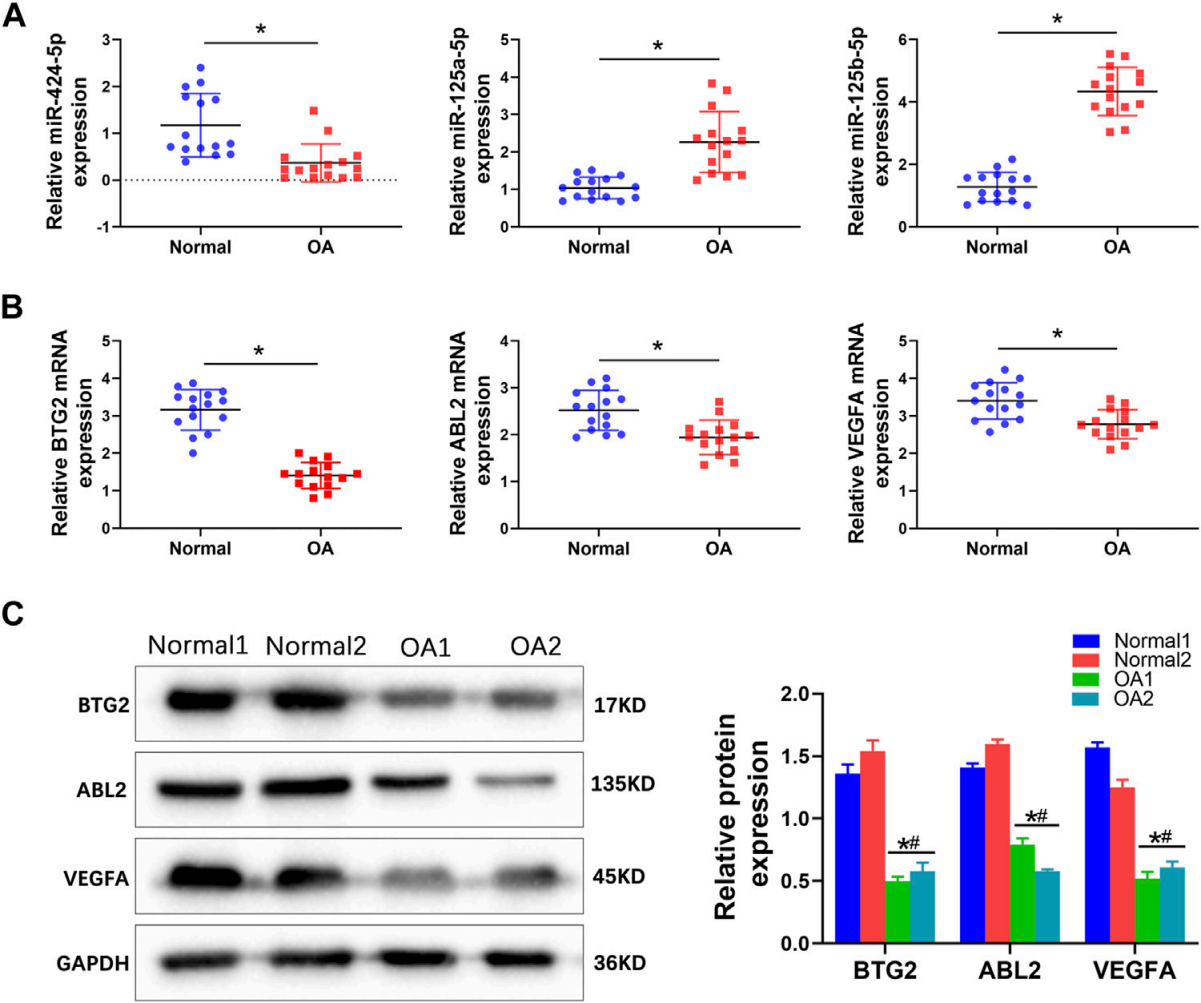
The validation of differentially expressed miRNAs and potential biomarkers in normal and OA knee cartilage tissues. **(A)** QRT-PCR was performed to detect the mRNA expression of miR-424-5p, miR-125a-5p, and miR-125b-5p in normal and OA knee cartilage tissues. **(B)** QRT-PCR was performed to detect the mRNA expression of BTG2, ABL2, and VEGFA in normal and OA knee cartilage tissues. **(C)** Western blotting analysis and relative quantification showing the protein expression of BTG2, ABL2, and VEGFA in normal and OA knee cartilage tissues. Values are mean ± SD, *p < 0.05 compared with the Normal 1 group; #*p* < 0.05 compared with the Normal 2 group.

QRT-PCR, Western blotting analysis, and immunofluorescent staining were performed to validate the expression levels of differentially expressed miRNAs, including miR-424-5p, miR-125a-5p, and miR-125b-5p and differentially expressed target genes, including BTG2, ABL2, and VEGFA in the ceRNA network. The qRT-PCR results showed that the expression levels of miR-424-5p were significantly downregulated, whereas the expression levels of miR-125a-5p and miR-125b-5p were remarkably upregulated in OA tissues compared with the normal group (Figure 7A). These results were consistent with the miRNA sequencing data. Furthermore, the mRNA expression levels of the target genes, including BTG2, ABL2, and VEGFA in OA tissues were all significantly decreased compared with the normal control group (Figure 7B). Similar effects on the expression of target genes were observed at protein levels. Western blotting analysis revealed an obviously decreased expression of BTG2, ABL2, and VEGFA in BMSCs in OA tissues (Figure 7C).

**FIGURE 8:**
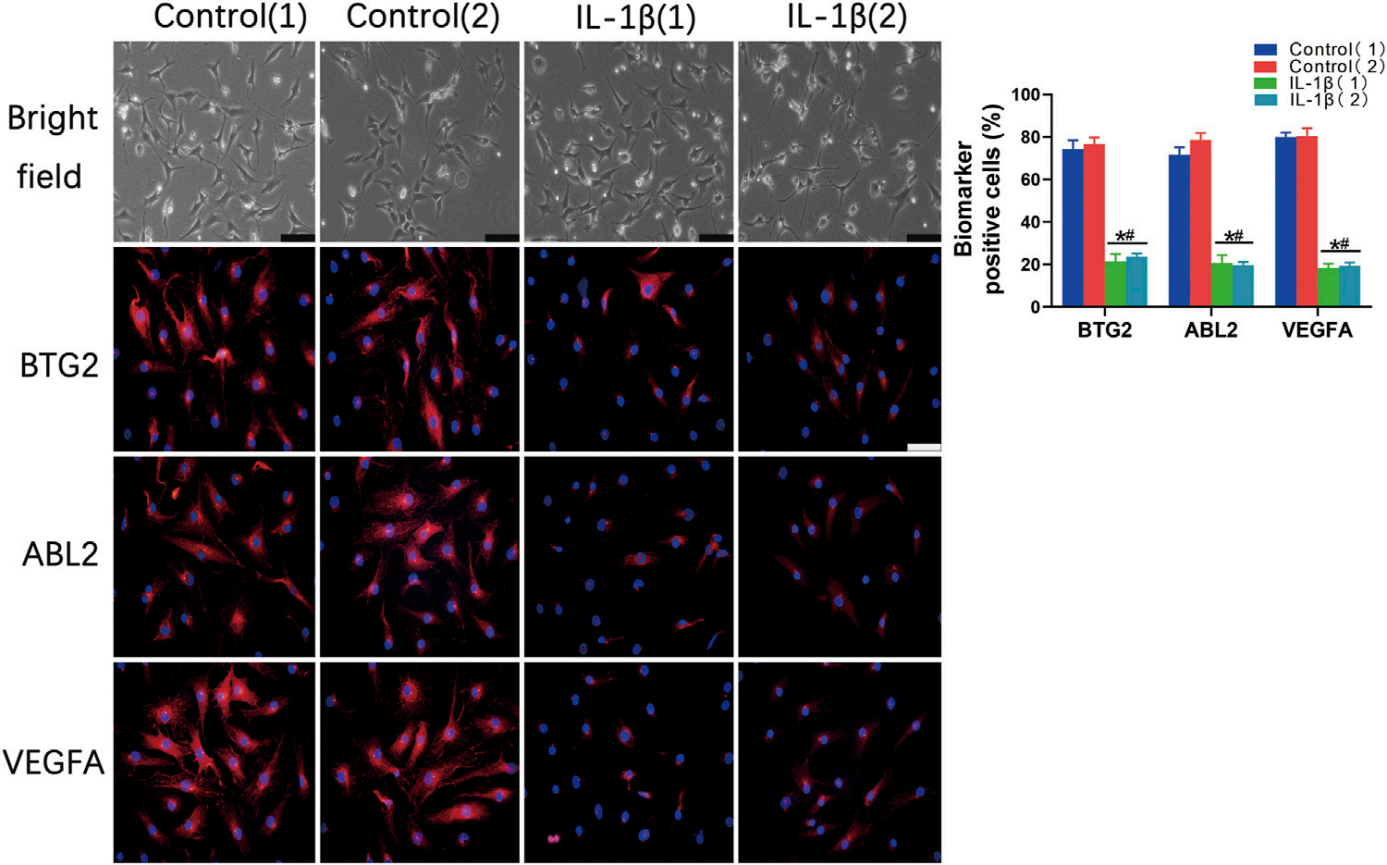
The validation of differentially expressed potential biomarkers in normal chondrocytes and IL-1β induced OA chondrocytes. Bright fields and immunofluorescent staining show the protein expression of potential biomarkers, including BTG2, ABL2, and VEGFA positive cells in normal chondrocytes and IL-1β induced OA chondrocytes. The percentage of BTG2, ABL2, and VEGFA positive cells of chondrocytes were also counted and evaluated. Values are mean ± SD. *p < 0.05, compared with the Control 1 group; #*p* < 0.05 compared with the Control 2 group.

The immunofluorescent staining results showed that the BTG2, ABL2, and VEGFA positive cell numbers were significantly decreased in the IL-1β induced OA chondrocytes in comparison to that of the control chondrocytes (Figure 8). Taken together, these results indicated that the abovementioned biomarkers exhibit consistent expression trends in OA conditions compared with that of the normal controls.

### MiR-125a-5p Negatively Regulates B-Cell Translocation Gene 2

**FIGURE 9:**
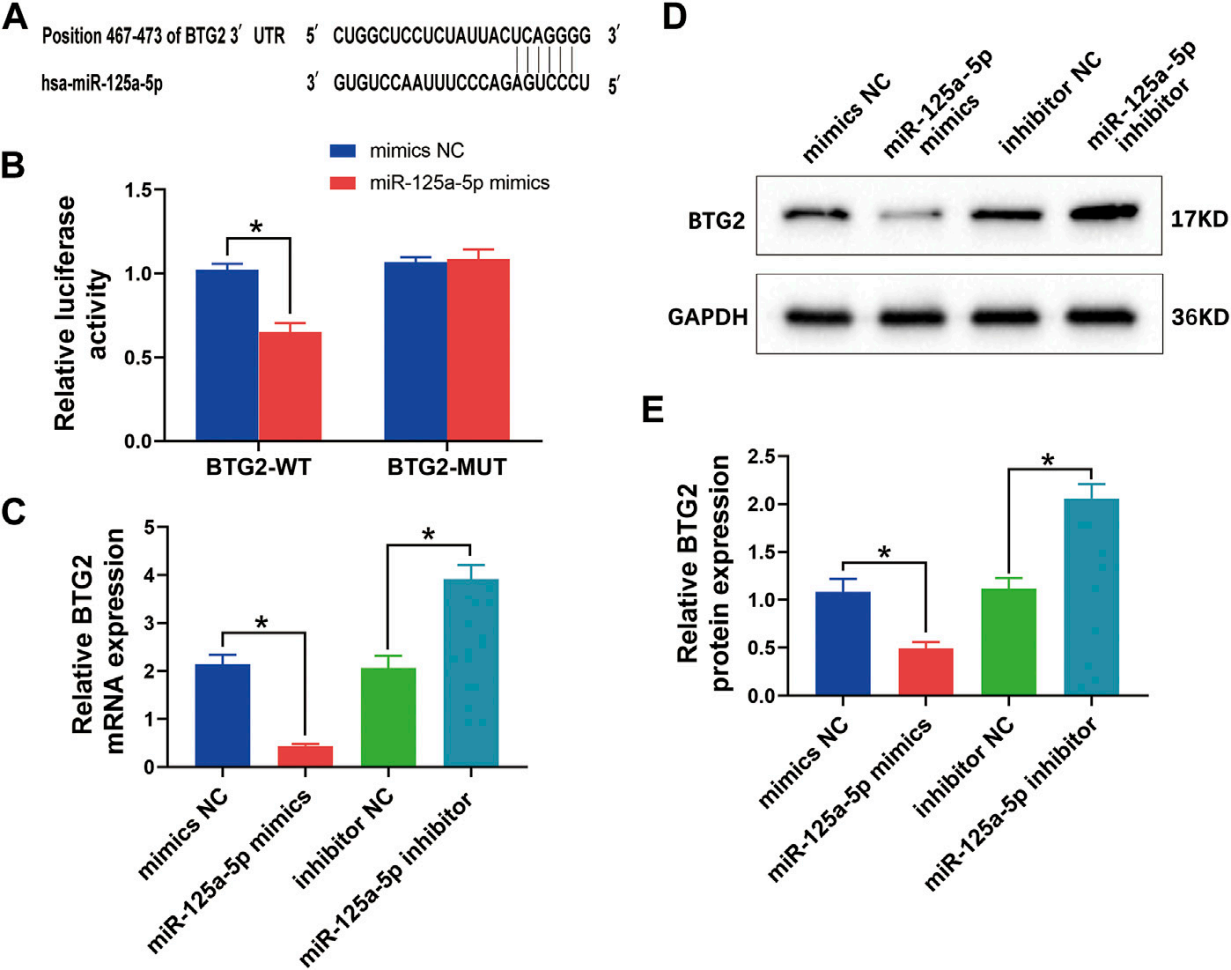
MiR-125a-5p negatively regulates BTG2. **(A)** The target gene BTG2 of miR-125a-5p was identified using the Targetscan software. **(B)** The relative luciferase activity between cells was compared after co-transfection of BTG2-WT/BTG2-MUT vectors with miR-125a-5p mimics and negative control. **(C)** QRT-PCR was performed to detect the mRNA expression of BTG2 in chondrocytes transfected with miR-125a-5p mimics, inhibitor, and their negative control. **(D** and **E)** Western blotting analysis and relative quantification showing the protein expression of BTG2 in chondrocytes transfected with miR-125a-5p mimics, inhibitor, and their negative control. GAPDH was used as the internal control. Values are mean ± SD. *p < 0.05.

The TargetScan software identified that miR-125a-5p could bind with the target sites in the 3′-UTR of BTG2, suggesting that BTG2 might be the target gene of miR-125a-5p (Figure 9A). To determine whether BTG2 was transcriptionally regulated by miR-125a-5p, the WT and MUT BTG2 3′-UTR segment containing the potential target sites for miR-125a-5p was constructed and cloned into the downstream region of a dual-luciferase reporter gene. MiR-125a-5p mimics had no effect on the luciferase activity of 293T cells with BTG2-MUT. However, the luciferase activity of the cells with NC + BTG2-WT was significantly higher than that of the cells with miR-125a-5p mimics + BTG2-WT. Thus, the miR-125a-5p mimics decreased the luciferase activity of cells with BTG2-WT (Figure 9B). These results suggested that miR-125a-5p could directly target BTG2.

To further elucidate the effect of miR-125a-5p on BTG2, chondrocytes were transfected with miR-125a-5p mimics, inhibitor, and their negative controls, respectively. The qRT-PCR analysis revealed a significant reduction of BTG2 mRNA level in chondrocytes treated with miR-125a-5p mimics compared with the control; conversely, miR-125a-5p inhibitor obviously enhanced BTG2 mRNA expression (Figure 9C). Similar effects on the expression of BTG2 were observed at protein levels as detected by Western blotting analysis (Figures 9D and E). These results suggested that BTG2 is negatively regulated by miR-125a-5p.

## Discussion

Taken together, our results demonstrate that BTG2, ABL2, and VEGFA were identified to be the central genes with good predictive performance, which were significantly correlated with immune cell infiltration in OA, reflected by declined aDCs and elevated contents of B cells, macrophages, neutrophils, and T helper cells. Moreover, anisomycin, MG-132, thapsigargin, and lycorine were predicted to be the candidate agents for potential treatments of OA awaiting further investigations. *In vitro*, the expression levels of differentially expressed miRNAs and potential biomarkers identified in the present study were consistent with the results obtained in normal or OA knee cartilage tissues and in chondrocytes. In addition, miR-125a-5p negatively regulated BTG2, suggesting the validation of miRNA–mRNA networks.

OA is a chronic degenerative joint disease which is frequently associated with severe joint damage and irreversible functional impairments. OA has affected 52.5 million Americans and generates 185 million economic burden each year (Metsios et al., 2008; Eakin et al., 2017; Pundole and Suarez-Almazor, 2020). It is estimated that there are around 654⋅1 million individuals (40 years and older) with knee OA in 2020 worldwide (Cui et al., 2020). OA has posed a threat to the quality of life of people and has emerged as a public health concern globally. However, at present, the early diagnosis and treatment of OA remain to be an important challenge. Various biomarkers show benefits in improving diagnosis, monitoring disease progression, and predicting prognosis. For the potential of guiding clinical therapies, biomarker identification for OA has attracted increasing attention. Over the last decade, the rise of epigenetics contributed to revealing the pathogenesis of autoimmune disorders and identifying promising candidate biomarkers (Shen et al., 2017; Oliviero et al., 2019; Tavallaee et al., 2020; Wan et al., 2021). Recent evidence has shown that miR-146a-5p is highly expressed in cartilage tissues and serum of OA patients when compared with that of the normal controls and is suggested as a biomarker for OA (Skrzypa et al., 2019). LncRNA PCGEM1 prostate-specific transcript (PCGEM1) is remarkably upregulated in late-stage OA compared with early OA, which has been suggested as an indicator for stratifying OA (Zhao and Xu, 2018). A recent bioinformatics analysis has revealed that the innate immunity-related gene of toll-like receptor 7 (TLR7) is differentially expressed in the synovial membrane, blood, and articular cartilage of OA patients compared with that of the normal controls, suggesting that TLR7 may serve as a potential biomarker for the early diagnosis and treatment of OA (Wang et al., 2020).

CeRNA network containing lncRNA–miRNA–mRNA interactions has been theorized to play an indispensable role in the progression of diseases and could help in exploring the pathogenesis of diseases and biomarker discovery. However, the study of the ceRNA network associated with OA is lacking. In this study, we constructed a ceRNA network and identified BTG2, ABL2, and VEGFA as the critical genes. BTG2, ABL2, and VEGFA were identified to be differentially expressed in synovial tissues of OA patients compared with normal controls based on three groups of mRNA datasets. WGCNA provides information on critical gene function modules and biomarkers for diseases (Tian et al., 2020). The above three genes were in the brown module that was significantly correlated with the clinical traits of OA. All these suggested that BTG2, ABL2, and VEGFA might play a key role in the pathogenesis of OA. In order to validate the critical role of BTG2, ABL2, and VEGFA in OA, we performed miRNA sequencing of synovial tissues of OA patients and normal controls. A total of 26 miRNAs were found to be differentially expressed in OA and the expression profile of miRNAs could clearly distinguish OA samples and normal control samples, indicating that the differentially expressed miRNAs might play important roles in OA. Then, with the mean of a bioinformatic tool for predicting miRNA–mRNA and lncRNA–miRNA interactions, a ceRNA network with DEGs in the brown module and miRNAs with differential expression was constructed. The central role of BTG2, ABL2, and VEGFA was highlighted based on the high node degree.

VEGFA, named vascular endothelial growth factor A, has been reported to be in association with OA (Guan et al., 2020). Zupan et al. (2018) reported that the high expression of VEGFA indicating cartilage or bone degeneration is associated with pre-osteoarthritis. Furthermore, overexpression of miR-140-5p alleviates knee osteoarthritis by downregulating VEGFA in a rat model (Liu et al., 2022). BTG2 was found to be a critical gene related to inflammation in OA. Recent evidence showed that BTG2 is a hub node in the ingenuity pathway network of facet joint OA, which proposed its function in mediating signaling pathways by affecting other genes (Chen et al., 2020). In addition, our ROC analysis indicated that BTG2, ABL2, and VEGFA showed a good potential predictive effect on OA. Thus, the three genes may serve as promising potential predictive biomarkers for OA.

It is well known that synovial inflammation plays a key role in the initiation of OA (de Lange-Brokaar et al., 2012). Recent evidence has shown that there is increased M1 macrophage infiltration and declined mast cell and neutrophil infiltration in OA samples (Yuan et al., 2021). The cartilage damage and synovitis in OA are characterized by abnormal cell proliferation and immune cell infiltration, including T cell and activated macrophages (Lopes et al., 2017). In this study, we found that the aDCs were significantly declined, while B cells, macrophages, T cell co-inhibition, T cell co-stimulation, and T helper cells were significantly elevated in the microenvironment of synovial tissues, which was agreed with the previous findings (Penkava et al., 2020; Van Raemdonck et al., 2020; Zhao et al., 2021). In addition, the expressions of BTG2, ABL2, and VEGFA were significantly correlated with the immune cell infiltration. Thus, the three critical genes may play key roles in the progression of OA by modulating the microenvironment of the articular cartilage.

Furthermore, thapsigargin was predicted to be a candidate small molecule that could reverse the OA process by targeting DEGs. It is reported that insufficient apoptosis of inflammatory cells contributes to the progression of OA, and enhanced apoptosis of chronic inflammatory cells in joints might be the therapeutic target for OA (Bar-Yehuda et al., 2009). Thapsigargin, as a well-known endoplasmic reticular Ca2+ ATPase inhibitor, has been widely used as an apoptosis inducer in tumor cells (Muthukkumar et al., 1995; Chen et al., 2016). Thus, thapsigargin could be suggested as the candidate drug for OA to enhance apoptosis in joints by targeting DEGs, including BTG2, ABL2, and VEGFA. Taken together, the expression levels of differentially expressed miRNAs, including miR-424-5p, miR-125a-5p, and miR-125b-5p and potential biomarkers, including BTG2, ABL2, and VEGFA were validated in normal or OA knee cartilage tissues and chondrocytes. The experimentally validated results are consistent with the bioinformatics analysis. Furthermore, *in vitro* experiments showed that miR-125a-5p negatively regulated BTG2, suggesting the existence of miRNA–mRNA networks in the pathogenesis of OA.

## Limitations

There were several limitations of our study. First, there is no *in vitro* or *in vivo* evidence in this study that the screened drugs are useful for the treatment of OA, but there is some literature evidence. Second, validation of potential biomarkers identified in the present study was not performed *in vivo* models of OA. Further experiments to validate the identified potential biomarkers and illustrate the pathological process and therapeutic targets of OA are therefore necessary.

## Conclusion

In summary, BTG2, ABL2, and VEGFA were differentially expressed genes in tissues of OA patients. BTG2, ABL2, and VEGFA were the central genes in the ceRNA network and played key roles in modulating immune cell infiltration in synovial tissues of OA. Moreover, anisomycin, MG-132, thapsigargin, and lycorine were predicted to be the candidate agents for OA. *In vitro*, the expression levels of differentially expressed miRNAs and potential biomarkers identified in the present study were consistent with the results validated in normal or OA knee tissues and in chondrocytes. BTG2, ABL2, and VEGFA could be regarded as potential diagnostic biomarkers for OA, which might guide the clinical therapy of OA.

## Data Availability

The original contributions presented in the study are included in the article/Supplementary Material; further inquiries can be directed to the corresponding authors.

## Abbreviations

OA, osteoarthritis; SOD2, serum superoxide dismutase 2; C2C, collagen type II cleavage neoepitope; CRP, C-reactive protein; Adam8, a disintegrin and metalloproteinase 8; Arg1, arginase 1; miRNAs, microRNAs; GEO, Gene Expression Omnibus; DEGs, differentially expressed genes; GO, Gene Ontology; KEGG, Kyoto Encyclopedia of Genes and Genomes; WGCNA, weighted gene co-expression network analysis; TOM, topological overlap matrix; lncRNA, long non-coding RNA; ROC, receiver operating characteristic; CMap, connectivity map; GSEA, gene set enrichment analysis; SVA, surrogate variable analysis; TNF, tumor necrosis factor alpha; IL-17, Interleukin-17; ceRNA, competing endogenous RNA; BTG2, B-cell translocation gene 2; ABL2, Abelson-related gene; VEGFA, vascular endothelial growth factor A; aDCs, activated dendritic cells; PCGEM1, PCGEM1 prostate-specific transcript; TLR7, toll-like receptor 7.
